# Sub-0.5 V Highly Stable Aqueous Salt Gated Metal Oxide Electronics

**DOI:** 10.1038/srep13088

**Published:** 2015-08-14

**Authors:** Sungjun Park, SeYeong Lee, Chang-Hyun Kim, Ilseop Lee, Won-June Lee, Sohee Kim, Byung-Geun Lee, Jae-Hyung Jang, Myung-Han Yoon

**Affiliations:** 1 School of Materials Science and Engineering, Gwangju Institute of Science and Technology, Gwangju 500-712, Republic of Korea; 2 Research Institute for Solar and Sustainable Energies, Gwangju Institute of Science and Technology, Gwangju 500-712, Republic of Korea; 3 School of Mechatronics, Gwangju Institute of Science and Technology, Gwangju 500-712, Republic of Korea; 4 School of Information and Communications, Gwangju Institute of Science and Technology, Gwangju 500-712, Republic of Korea

## Abstract

Recently, growing interest in implantable bionics and biochemical sensors spurred the research for developing non-conventional electronics with excellent device characteristics at low operation voltages and prolonged device stability under physiological conditions. Herein, we report high-performance aqueous electrolyte-gated thin-film transistors using a sol-gel amorphous metal oxide semiconductor and aqueous electrolyte dielectrics based on small ionic salts. The proper selection of channel material (i.e., indium-gallium-zinc-oxide) and precautious passivation of non-channel areas enabled the development of simple but highly stable metal oxide transistors manifested by low operation voltages within 0.5 V, high transconductance of ~1.0 mS, large current on-off ratios over 10^7^, and fast inverter responses up to several hundred hertz without device degradation even in physiologically-relevant ionic solutions. In conjunction with excellent transistor characteristics, investigation of the electrochemical nature of the metal oxide-electrolyte interface may contribute to the development of a viable bio-electronic platform directly interfacing with biological entities *in vivo*.

Sol-gel derived amorphous metal-oxide (MO_x_) semiconductors have been intensively studied for a variety of applications including displays^1^,^2^,^3^,^4^, sensors^5^,^6^, memories^7^,^8^, and photovoltaics^9^,^10^,^11^ with a recent emphasis on flexible transparent electronics^12^,^13^,^14^,^15^,^16^. In certain areas of device applications, this class of electronic materials can now compete with or outperform silicon due to their unique attributes, for instance, low-temperature and solution processability^12^,^13^,^14^,^15^,^16^,^17^, high optical transparency^14^,^18^, and film uniformity^3^, in addition to excellent electrical properties. Despite the in-depth understanding of material and device properties, MO_x_-based electronic materials still have unexplored potential for unconventional device applications such as *in vivo* biochemical sensors and implantable human-machine interfaces which gradually gain technological as well as social interests^19^,^20^,^21^,^22^,^23^,^24^,^25^,^26^,^27^. Nonetheless, water-stable high-performance electronics employing aqueous salt environments has not been developed and charge transport at the water-metal oxide interface mimicking physiological salt conditions in human body has not been systematically investigated yet.

Electrolyte-gated thin-film transistor (EGTFT) is a type of thin-film transistors in which various forms of electrolyte-containing dielectrics are employed as a gate-insulating medium. The areal capacitances of typical dielectric materials used in conventional TFTs are in the range of 0.005 ~ 0.5 μF cm^−2^, which are determined and thus limited by their thickness and dielectric constant^28^. On the other hand, the electrolyte-based electrical double layers (EDLs) exhibit exceptionally large areal capacitance typically larger than 10 μF cm^−2^ with no virtual dependence on dielectric film thickness^29^,^30^,^31^,^32^,^33^,^34^. In a simplified model, an EDL is analogous to a conventional two parallel-plate capacitor where highly-dense surface charges on solid electrode and oppositely-charged ions in liquid electrolyte are aligned at the phase interface with an angstrom-scale spacing^35^,^36^,^37^. This feature underlines efficient carrier accumulation at the active channel and low-voltage operation in a variety of EGTFTs^29^,^30^,^31^,^32^,^33^,^34^,^35^,^36^,^37^.

Frisbie^29^, Dasgupta^30^, Iwasa^31^, and Zaumseil^32^ groups successfully demonstrated low-voltage MO_x_ EGTFTs using ionic liquids or polymeric electrolytes although some of these TFTs exhibit substantial performance degradation during prolonged operation in ambient conditions. Despite the abovementioned potential of water-stable high-performance electronics, there have been very few studies on MO_x_ EGTFTs stably operating in aqueous salt solutions due to degradation in oxide material itself or device^33^,^34^. In parallel, a similar type of water-compatible TFTs or organic electrochemical transistors (OECTs) employing organic semiconductors^21^,^22^,^23^,^24^ or conducting polymers^25^,^26^,^27^ (e.g., PEDOT:PSS) have been under intensive research for eventual applications in the biomedical system. However, typical OECTs in aqueous ionic solutions exhibit several drawbacks including relatively small current on-off ratios, high leakage currents, slow dynamic responses, and narrow operation voltage window which are attributed to the distinct operation mechanism based on ion permeation and consequent electrochemical reactions at the polymer-electrolyte interface^20^.

In this study, we report low-voltage EGTFTs using a sol-gel derived amorphous indium gallium zinc oxide (IGZO) semiconductor and various aqueous solutions of common ionic salts. The DC and frequency-dependent impedance characteristics of the resultant water-based EGTFT devices are comprehensively investigated, particularly focusing on the influence of various aqueous electrolyte conditions on the charge accumulation at the water-metal oxide interface. With the proper passivation of non-channel areas and judicious selection of electrolyte solution, the optimized structure of EGTFTs and their device characteristics are discussed in the following from the perspective of potential usage under physiologically relevant ionic conditions for the future bionics application.

A schematic of EGTFT using sol-gel indium-gallium-zinc-oxide (IGZO) and aqueous salt solutions is illustrated in Fig. 1a with the actual photographic image (Fig. 1b). First, unlike typical sol-gel metal oxide TFTs which are constructed in a bottom-gate top-contact configuration, a top-gate bottom-contact configuration was adopted in this study so that the final device structure can support a liquid form of gate dielectric material on top of channel layer and avoid the direct contact between source/drain electrodes and electrolyte solution. Moreover, Au/Cr patterns and a gold-coated tungsten tapered tip (shank diameter ~0.5 mm) were used as source/drain contact to attain efficient charge injection from oxide-free metal surface to semiconducting channel and gate electrode to minimize faradaic gate leakage current via effective polarization at the metal-electrolyte interface, respectively. Subsequently, UV-curable epoxy passivation layer was patterned on top of the IGZO channel in order to avoid electrical short between source/drain lead lines and an aqueous salt solution as well as to define a reservoir well for salt-containing liquid.

Figure 1c shows the representative transfer curves of IGZO EGTFTs operated in DI water and various salt solutions. All the transfer curves exhibit typical n-type characteristics indicated by charge accumulation at positive gate biases (*V*_G_). DI water-gated TFTs show impressive device characteristics, i.e. operation voltages below 0.5 V, current on-off ratios of 10^7^, threshold voltage of 0.15 V, and subthreshold swing of 74 mV dec^−1^, even without additional electrolyte in water. More remarkably, high transconductance (∆*I*_D_/∆*V*_G_) above 0.5 mS and large on-current level in the order of 0.1 mA (*V*_G_, *V*_D_ = 0.5 V) verify efficient field-effect current modulation and excellent current driving capability in this TFT structure, respectively. Considering that there are no faradic peaks in cyclic voltammograms within the ±0.5 V (Figure S1), the low off-state current and thus very high on-off current ratio benefit from the electrochemical inactivity at the IGZO-water interface (*vide infra*) unlike OECT where ion penetration into the channel layer plays an important role in channel current modulation^20^.

To investigate the effect of ionic species on aqueous EGTFT characteristics, aqueous solutions of 1.0 M sodium chloride (NaCl), potassium chloride (KCl), and potassium bromide (KBr) were used for gate electrolyte solutions. Note that Na^+^, K^+^, and Cl^−^ ions were selected because they are the most abundant monovalent ions in human body fluid^38^ and all of the used inorganic salts are expected to be fully dissociated in water. By switching the dielectric medium from DI water to aqueous ionic solutions, the corresponding TFT transfer plots exhibit the improved figure-of-merits as revealed by enhanced transconductances, close-to-zero threshold voltages, reduced subthreshold swings, and negligible hysteresis (Fig 1d–f, and S2). The extents of performance enhancement at the same salt concentration were very similar to each other, which indicates that extra ions added into DI water contribute to more effective charge accumulation at the channel regardless of the type of cation or anion. The origin of abnormal off-state current behaviour in EGTFT employing aqueous KBr dielectric remains speculative and needs to be further investigated. The operational stability of EGTFTs was examined by periodically applying positive and negative gate biases (*V*_G_ = +0.3 V [on] or −0.3 V [off] when *V*_D_ = 0.5 V and Δ*t* = 1 s) over 1000 cycles. As shown in Fig. 1g, the recorded time-varying drain currents remained close to the initial value, indicating that there was no significant oxide film dissolution or device performance degradation for ~10^3^ s, which corresponds to the minimum settle-down time required in the potential application for sensing very small amount of biological analytes^39^.

Subsequently, the electrical characterization of IGZO-EGTFTs with different KCl concentrations was performed to investigate the effects of small ion concentration on the transconductance (g_m_) of the devices. As shown in Fig. 2a,b, the on-state channel currents in transfer and output curves increased with higher concentration of KCl solutions. Clearly-defined linear and saturation regimes in the output plots were observed with negligible parasitic leakage. The statistical analysis of maximum transconductance shows that the enhancement of channel current became more prominent with the higher KCl concentration in gate dielectric media (Fig. 2c). In contrast, threshold voltage (V_T_) and subthreshold swing (S.S.) followed the different trend; once these metrics slightly decreased upon introducing ionic salts in DI water dielectric, they remained almost invariant regardless of the actual ionic concentration increase (Fig 2d,e).

To further investigate the correlation between device characteristics of aqueous IGZO EGTFTs and electrochemical properties at the IGZO-water interface, we performed electrochemical impedance spectroscopy (EIS) on coplanar-type Au/electrolyte/Au and Au/electrolyte/IGZO/Au devices. Then, the experimental results were analysed using an equivalent circuit model. As shown in Bode plot, the increased ionic concentration pushed the capacitive plateau toward higher cut-off frequency by the reduced series resistance (Rs) of bulk electrolyte solution (Fig. 3a). The concomitant shift of the real-axis intercept in the Nyquist plot became more prominent with the increased ionic concentration for the same reason (Fig. 3b). Furthermore, the absence of distinguishable circles as well as almost vertical straight lines in Nyquist plots confirms that parallel resistance component (Rp) should be very high due to minimized faradaic current or inefficient charge transfer reactions on the oxide surface. All the EIS measurements indicate that these aqueous electrolyte devices are mainly dominated by the capacitive behaviour of EDL while the bulk solution resistance is very small and the IGZO surface is electrochemically inert. Note that such a simple phase angle behaviour close to −90 degrees over a large frequency range is in clear contrast to the ZnO EGTFTs using ionic liquids showing complicated frequency responses^31^,^32^. Accordingly, the areal capacitance of EDL (*C*_EDL_) at the Au-electrolyte and IGZO-electrolyte interfaces was calculated directly from the imaginary component (Z”) in the measured EIS data (See Supplementary Information for the detailed information, Figure S3 and S4). All the extracted metrics at different ion concentrations and the electrical parameters of corresponding EGTFTs are listed in Table 1. *C*_EDL_ of the Au surface increased up to 30 μF cm^−2^ with higher ionic contribution, however, the extracted TFT mobilities remained almost constant (~9 cm^2^/Vs) regardless of ion type and concentration in aqueous dielectric media. Interestingly, these mobility values are comparable to those of sol-gel MOx TFTs using different solid dielectrics reported in the previous literature^12^,^13^,^14^,^15^,^16^, verifying that the aforementioned high transconductance in aqueous IGZO EGTFTs stems from the efficient charge carrier accumulation mediated by the high EDL capacitance without damage in the semiconducting channel even in the presence of water and small ionic species.

As a proof of time-varying device application, the resistor-loaded inverter was electrically characterized. Fig. 4a shows output voltage (*V*_OUT_) of the given inverter in response to the input voltage (*V*_IN_) at different KCl concentrations (*V*_DD_ = 0.5 V). The maximum voltage gain (d*V*_OUT_/d*V*_IN_), 3.77 was measured at *V*_IN_ near 0.23 V (Figure S5). Furthermore, the increased ionic strength enabled steeper voltage turn-down, which is predominantly attributed to the enhanced current driving capability. On the other hand, the shift in voltage transition point corresponds to that of the threshold voltage. Fig. 4b shows the representative dynamic inverter responses to the 10-Hz square-wave input signal. The observed maximum operation frequency was several hundred Hz while their operational stability remained up to 8 hours (Figure S6 and S7). The rising edges of the *V*_OUT_ curves show a clear spread of response dynamics and, therefore, the signal delay parameter (*τ*) was extracted at different ion concentrations. By using an exponential rising model, the first positive half-period (*t* from 0 to 50 ms) was fitted into the equation (1).,


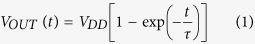


where *V*_OUT_ is output voltage, *V*_DD_ is driving voltage, and *τ* is characteristic time constant which corresponds to the time required for *V*_OUT_ to reach 64% of *V*_DD._ The calculations presented in Fig. 4c confirm the validity of the exponential model by the linear behaviours from all data sets and therefore each slope is unambiguously extracted and, in turn, converted to *τ* as a function of KCl concentration (Fig. 4d). With increasing salt concentration, characteristic time constant decreased, and, once the salt concentration is above 1.0 M, this number was ultimately converged to <2 ms. The faster response of inverter at higher ionic concentration is in full agreement with the static transistor behaviour and EIS results presented above. Since the IGZO channel performance is virtually invariant to the ionic concentration in dielectric media, aqueous electrolytes are solely responsible for the dynamic response shown in Fig. 4d. The increased ion concentration induces the reduction in solution resistance and the increase in interfacial capacitance (Fig. 3), and, thereby, enables faster charging/discharging in response to alternating *V*_IN_ pulses, which suggests the dramatic decrease in time constant (τ) in the presence of aqueous electrolytes.

So far, we demonstrated that aqueous IGZO EGTFTs support excellent charge transport effectively modulated by both static and dynamic gate biases via EDL-mediated capacitive effect. To our knowledge, these EGTFTs are located in the top rank of the figure-of-merit table (i.e., operation voltage, transconductance, current on-off ratio) in comparison with various types of EGTFTs using other types of gate dielectric electrolyte (Table S1 and Figure S8). Compared with fully-dissociated pure ionic liquid, aqueous ionic solutions with 0.1–2.0 M concentration contain much smaller number of solvated ions (e.g., Na^+^, K^+^, and Cl^−^), however, they exhibit equivalent or higher EDL capacitance, possibly, due to the smaller size and higher mobility of salt ions leading to more compact EDL formation. Furthermore, the improved TFT performance compared with OECTs using the same type of aqueous salt solutions as a dielectric medium is attributed to higher carrier mobilities of metal oxide semiconductor and the absence of ion-mediated electrochemical reactions at the water-IGZO interface.

Finally, to examine the compatibility of aqueous IGZO EGTFTs with physiological salt and pH conditions, a phosphate buffered saline (PBS) solution was employed for gate dielectric medium. PBS is an isotonic buffer solution (pH ~7.4) where osmolality and ion concentrations (e.g., Na^+^, K^+^, Cl^-^, phosphate, and etc.) were set to those inside human body, and, therefore, is conventionally used as a model solution for various cell viability and protein handling experiments. Fig. 5a,b show representative transfer and output curves of IGZO EGTFTs using PBS dielectric. These devices stably functioned under physiologically relevant ionic concentrations and exhibited very high transconductances (~1.0 mS) and exceptionally large on-off current ratios (>10^7^) with small hysteresis (Table 1). This observation also supports that both IGZO EGTFT device itself and operation in water were not significantly affected by isotonic salt condition and phosphate ion existence, which is the most abundant anion in intracellular fluid of human. Finally, the alternating gate bias test (*V*_G_ = +0.3 *V* [on] or −0.3 V [off], *V*_D_ = 0.5 V, Δ*t* = 1 s) and the statistical analysis of TFT characteristics confirm the prolonged (>10^3^ s) operational stability unlike thin silicon or other metal oxide devices which are slowly dissolved under similar aqueous salt conditions (Fig. 5c-f)^32^,^40^,^41^.

In summary, we demonstrate very stable high-performance electrolyte-gated thin-film transistors using sol-gel amorphous IGZO semiconductor and aqueous salt dielectrics (KCl, NaCl, KBr ions in water, and PBS solution) showing sub-0.5 V operation, high on-off current ratio, excellent transconductance, and clear pinch-off behaviour. We expect that such an excellent electrical performance and long-term operational stability (up to 8 hrs) of the aqueous IGZO EGTFTs may contribute to the development of future human-friendly bio-electronics such as reusable/*in-vivo* biochemical sensors and implantable bionics.

## Methods

### Preparation of IGZO precursor solution

Indium nitrate hydrate (In(NO_3_)_3_·x(H_2_O)), gallium nitrate hydrate (Ga(NO_3_)_3_·x(H_2_O)), and zinc acetate dehydrate (Zn(CH_3_COO)_2_·2(H_2_O)) were purchased from Sigma-Aldrich. All precursors were dissolved in a 2-methoxyethanol solvent with molar concentrations of 0.085:0.0125:0.0275 for indium, gallium, and zinc precursors. The solution was vigorously stirred for 12 h at 75 °C before use.

### Preparation of ionic salt solutions

Distilled water was prepared by Water Purification System (Human RO 280, DAIHAN) showing 3 μS/cm of resistivity and 7.3 of pH value. KCl, NaCl, and KBr powders were purchased from Sigma-Aldrich. Each ionic salt was dissolved in purified DI water and stirred for 1 h before use. PBS solution was purchased from Gibco-BRL (Gaithersburg, MD, USA).

### EGTFT device fabrication

TGBC TFTs and coplanar capacitors were fabricated on pre-cleaned quartz substrates. Gold electrodes were patterned by the conventional photolithography, metal evaporation, and lift-off processes with the channel width and length defined as 200 and 20 μm, respectively. An IGZO solution was filtered through a 0.2 μm PTFE syringe filter and spun on Au-patterned substrates at 3500 rpm for 30 s. After annealing on a hot plate at 350 °C for 1 h, the resultant oxide films were photolithographically patterned for individual channel isolation. SU-8 photoresist (4 μm thick) was spin-coated (5000 rpm, 40 s), annealed, and patterned for passivation. Water or ionic salt solutions were added into the devices while the gate-contact Au probe was immersed for device measurement.

### Electrical characterisation

TFT characterization and EIS analysis were performed at room temperature in ambient conditions with Keithley 4200 semiconductor parametric analyzer and potentiostats/galvanostats (Autolab, Holland), respectively.

## Additional Information

**How to cite this article**: Park, S. *et al.* Sub-0.5 V Highly Stable Aqueous Salt Gated Metal Oxide Electronics. *Sci. Rep.* **5**, 13088; doi: 10.1038/srep13088 (2015).

## Supplementary Material

Supplementary Information

## Figures and Tables

**Figure 1:**
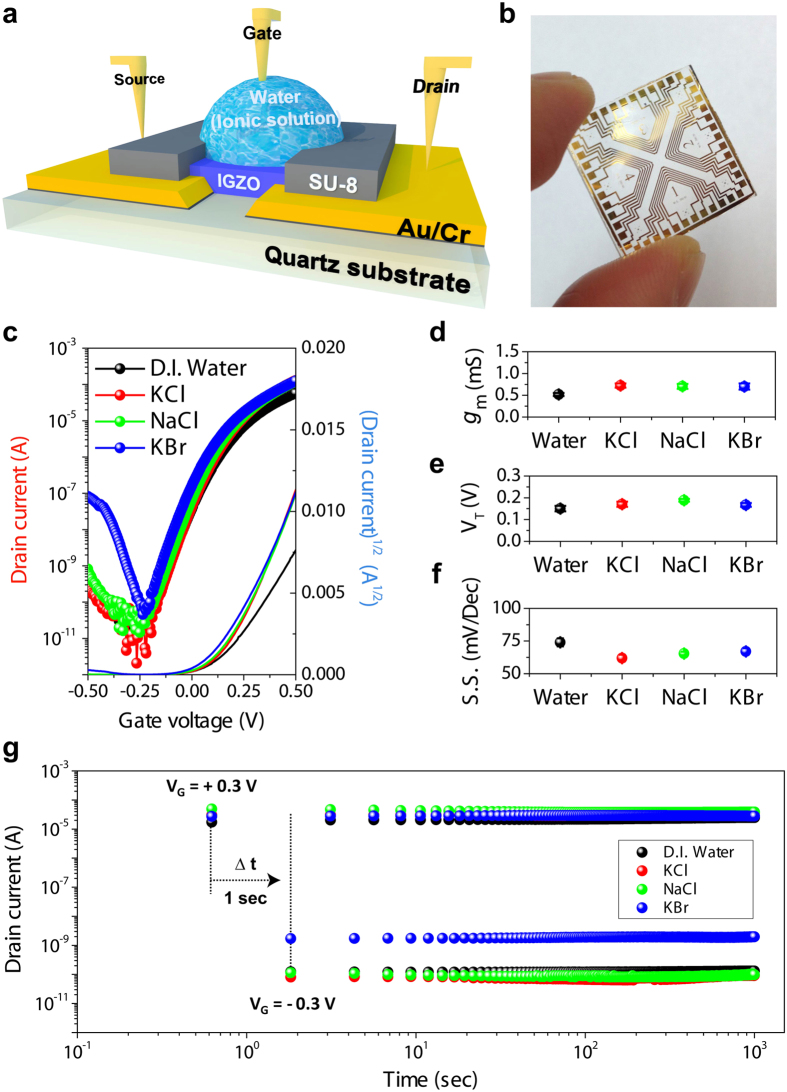
Electrolyte-gated IGZO thin film transistors (IGZO-EGTFTs) using various aqueous dielectrics. (**a**) A schematic of aqueous salt EGTFT structure composed of a quartz substrate, source and drain Au/Cr electrode patterns, an IGZO semiconducting layer, an SU-8 passivation well for electrical isolation and solution reservoir containing pure water or salt solutions as dielectric media (from bottom to top). (**b**) A photographic image of completed IGZO-EGTFT device arrays on a quartz substrate. (**c**) Representative transfer curves of IGZO EGTFTs using various solutions at *V_D_* = +0.5 V. (Black, red, green, and blue lines correspond to DI water, KCl, NaCl, and KBr salt solutions, respectively). Average values of (**d**) maximum transconductance (defined as *g*_*m*_ *=* *∆I*_*D*_/*∆V*_*G*_), (**e**) threshold voltage, and (**f**) subthreshold swings (S.S.); error bars denote standard deviations over 10 device measurements. (**g**) Drain current versus time (log scales) plot obtained every 1 s at +0.3 V and −0.3 V of alternating gate biases with +0.5 V of a constant drain voltage over 10^3^ cycles.

**Figure 2:**
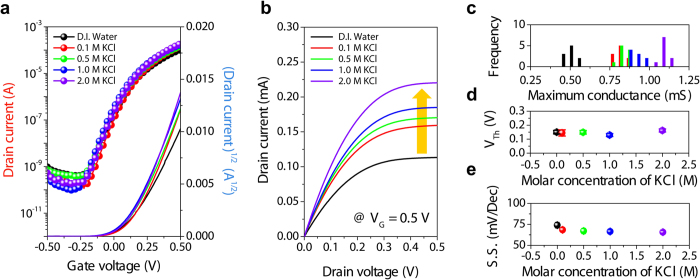
Electrical characteristics of IGZO-EGTFT at different salt concentrations. Representative (**a**) transfer (at *V*_*D*_ = 0.5 V) and (**b**) output curves (at *V*_*G*_ = 0.5 V) of IGZO-EGTFTs employing KCl solutions with various concentrations (from 0 to 2.0 M). (**c**) Statistical distributions of maximum transconductance extracted from 50 different IGZO-EGTFT devices. Average values of (**d**) threshold voltage, and (**e**) subthreshold swing; error bars denote standard deviations over 10 devices.

**Figure 3:**
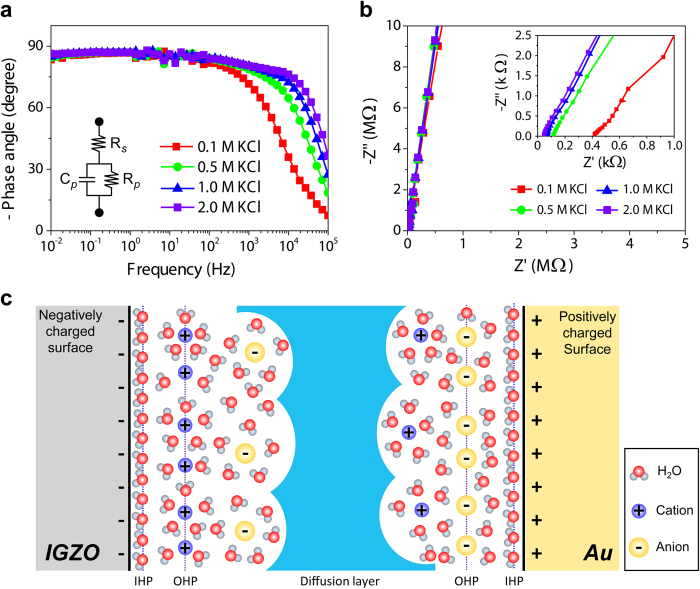
Electrochemical impedance analysis of IGZO/Electrolyte/Au structure. (**a**) Phase-frequency plots for Au/salt-solution/IGZO/Au structures. The inset shows an equivalent circuit model composed of series and parallel resistances and a capacitor. (**b**) Nyquist plots at high-frequency regime showing the real (Z’) and imaginary (Z”) parts of impedance. The near-vertical straight lines indicate that there is almost no faradaic reaction on oxide surface. The inset shows magnified Nyquist plots at low impedance region indicating that series resistance or bulk solution resistance decreased with increased KCl concentration. (**c**) An illustration of EDLs formed at IGZO and Au surface interfacing with an aqueous salt solution. The negatively (positively) charged surface exhibits an inner Helmholtz plane (IHP) of compact water layer and an outer Helmholtz plane (OHP) of diffusive hydrated cation (anions) layer.

**Figure 4:**
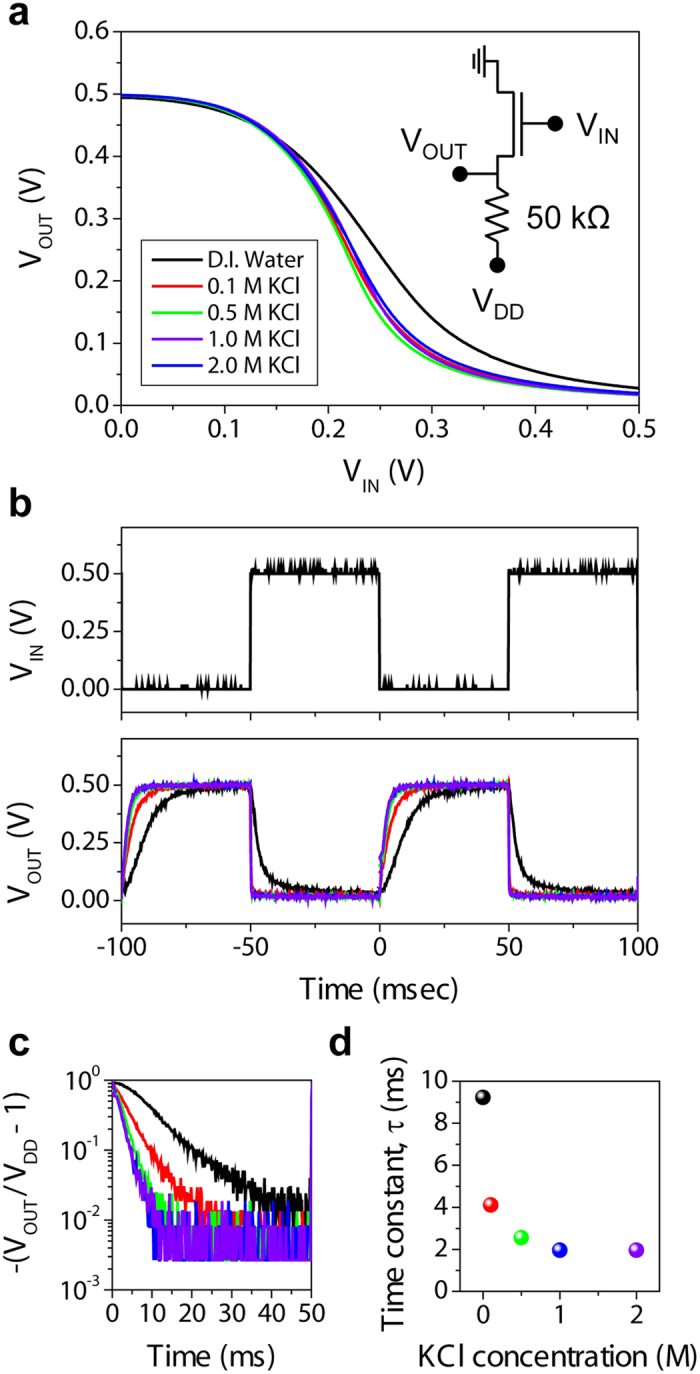
Resistor-loaded EGTFT inverters operated under aqueous salt conditions. (**a**) Static behaviours of resistor (50 kΩ) loaded IGZO EGTFT (*W*/*L* = 200 μm/20 μm) inverters with different aqueous KCl concentrations (from 0.1 to 2.0 M) with *V*_*DD*_ set to 0.5 V. (**b**) Dynamic output characteristics of EGTFT inverters in response to the square wave input (10 Hz) depending on different salt concentrations. (**c**) Semi-log plots of [−(*V*_*OUT*_/*V*_*DD*_ – 1)] versus time drawn as a validity proof of exponential rise behaviour. (**d**) Extracted time constant as a function of KCl concentration. Represented data sets are labelled as follows; black: D.I. water, red: 0.1 M, green: 0.5 M, blue: 1.0 M, violet: 2.0 M of KCl solution.

**Figure 5:**
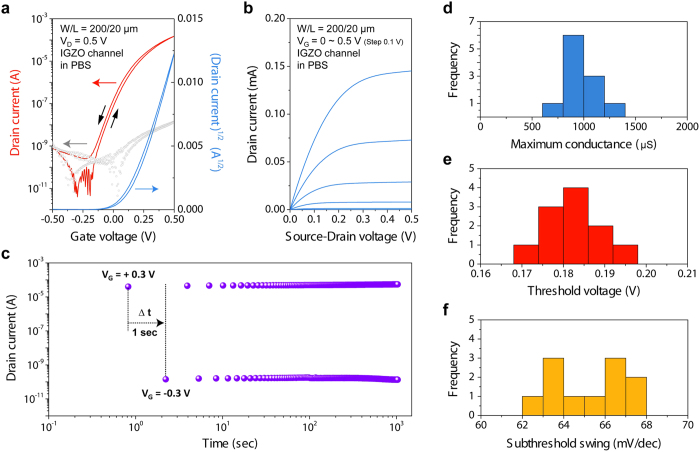
Electrical characteristics of IGZO-EGTFTs operated in a PBS solution. (**a**) Representative bidirectional sweep of transfer curves (red), square-root of channel current (blue), and gate leakage current (gray) at V_D_ = +0.5 V. (**b**) output curves at different gate biases (0 to +0.5 V with a 0.1 V step). (**c**) Drain current versus time (log scales) measured at every 1 s with alternating gate biases of +0.3 and −0.3 V and a constant drain bias of +0.5 V of over 10^3^ cycles. Statistical distributions of (**d**) maximum transconductance (g_m_,_max_), (**e**) threshold voltage (V_Th_), and (**f**) subthreshold swing (S.S.).

**Table 1:** A summary of electrical characteristics of IGZO EGTFTs under various aqueous dielectric conditions.

Semiconductor	Dielectric medium	Capacitance [μF/cm^2^]	μ_Sat_ [cm^2^V^−1^s^−1^]	V_Th_ [V]	g_m,max_ [mS]	I_on/off, Max_
IGZO	Water	14.63	10.15	0.15	0.519	4.5 × 10^6^
	KCl (0.1 M)	25.52	9.22	0.14	0.817	2.2 × 10^7^
	KCl (0.5 M)	27.87	8.74	0.15	0.844	1.2 × 10^7^
	KCl (1.0 M)	28.35	7.90	0.17	0.724	4.8 × 10^7^
	KCl (2.0 M)	30.12	10.82	0.16	1.086	1.4 × 10^7^
IGZO	KCl (1.0 M)	28.35	7.90	0.17	0.724	4.8 × 10^7^
	NaCl (1.0 M)	28.46	8.15	0.19	0.705	8.9 × 10^7^
	KBr (1.0 M)	26.01	8.15	0.17	0.700	2.7 × 10^6^
IGZO	PBS	30.12	10.21	0.18	0.968	2.1 × 10^7^

Field-effect mobilities were measured in a saturation regime [at V_D_ = 0.5 V, W/L = 200 μm/ 20 μm]. All data were averaged over 10 devices.
