# Supplementary material for: Sub-0.5 V Highly Stable Aqueous Salt Gated Metal Oxide Electronics
Source: Sci Rep. 2015 Aug 14;5:13088. doi: 10.1038/srep13088 (PMC4642510; doi:10.1038/srep13088)
Supplement: Supplementary Information [file srep13088-s1.doc]

**Supplementary Information**

**Sub-0.5 V Highly Stable Aqueous Salt Gated**

**Metal Oxide Electronics**

Sungjun Park, Se-Yeong Lee, Chang-Hyun Kim, Ilseop Lee, Won-June Lee, Sohee Kim, Byung-Geun Lee, Jae-Hyung Jang, and Myung-Han Yoon*

Corresponding author. Prof. Myung-Han Yoon

E-mail: mhyoon@gist.ac.kr (M-H.Y.)

**This “Supplementary Information” includes:**

Figs. S1 to S8

Table S1

References S1 to S19

**Table of Contents**

**1. Experiment**

1.1. Materials and precursor solution synthesis

1.2. Fabrication of electrolyte gated thin film transistors (EGTFTs)

1.3. Fabrication of M-I-M devices for impedance analysis

1.4. Electrical characterisation

**2. Cyclic voltammetry measurements**

**3. Characterisation of EGTFTs under potassium chloride (KCl) solutions**

**4. Impedance analysis**

**5. Inverter circuits operation in aqueous solution**

5.1. Inverter behaviour operated in aqueous solution depending on concentration

5.2. Signal responses depending on pulse frequency

5.3. Operational stability of circuits in aqueous solution

**6. Comparison of TFT parameters of reported water gated transistors**

**7. References**

**1. Experiment**

*1.1. Materials and precursor solution synthesis*

Indium nitrate hydrate (In(NO3)3•x(H2O)), gallium nitrate hydrate (Ga(NO3)3•x(H2O)), and zinc acetate dehydrate (Zn(CH3COO)2•2(H2O)) were purchased from Sigma-Aldrich. All precursors were dissolved in 2-methoxy ethanol solvent with a molar concentration of 0.085:0.0125:0.0275 for indium, gallium, and zinc precursors (which were synthesised and deposited using a procedure similar to that described in a previous report^S1^). After preparation, the solution was vigorously stirred for 12 h at 75 °C before use. The experiments were performed after the solution had been stored for 30 min at room temperature. For synthesis of the ionic solutions, potassium chloride, sodium chloride, and potassium bromide (denoted as KCl, NaCl, and KBr) were purchased from Sigma-Aldrich and used without further purification. Deionized (DI) water used in this study is purified using a Water Purification System (Human RO 280). The properties of water include a resistivity of 3 µS/cm and a pH value of 7.3. All ionic solutions were dissolved in purified DI water and stirred for 1 h before use. PBS solution was purchased from Gibco-BRL (Gaithersburg, MD).

*1.2. Fabrication of electrolyte gated thin film transistors (EGTFTs)*

Quartz wafers were cleaned by using DI water, acetone, and isopropanol and then dried by nitrogen. All substrates are 2 cm square sized. The top gate–bottom contact (TGBT) configuration was chosen in this study. First, Cr (50 Å)/Au (1000 Å) source-drain electrodes were thermally evaporated on the quartz substrates (base pressure: ~ 3×10^−6^ torr, deposition rate: ~ 0.2 Å/s) and subsequently patterned by conventional photolithography. The indium-gallium-zinc-oxide (IGZO) solution was spin coated at 3500 rpm for 30 s, followed by a thermal annealing step on a hot plate at 350 °C for 1 h. To reduce the parasitic and additional leakage current, the IGZO thin films were patterned by PR-photolithography and wet etching (LCE-12). For the electrical isolation, epoxy-based SU-8, a well-known bio-compatible material, was used for passivation of the electrodes outside of the active semiconducting layer regions. SU-8 photoresist was spin-coated at 5000 rpm for 40 s and prebaked at 65 °C for 2 min and at 95 °C for 6 min to remove the solvent and anneal the SU-8 film. Then, SU-8 film was exposed to UV light for 35 s in hard contact mode. We used SU-8 developer for 2 minutes as a PR developer. Thickness of the patterned SU-8 film was approximately 4 μm.

*1.3. Fabrication of M-I-M devices for impedance analysis*

Quartz wafers were cleaned by using deionized water, acetone, and isopropanol and then dried by nitrogen. All substrates are 2 cm square sized. Cr (50 Å)/Au (1000 Å) source-drain electrodes were thermally evaporated on the quartz substrates (base pressure: ~ 3×10^−6^ torr, deposition rate: ~ 0.2 Å/s) and subsequently patterned by conventional photolithography. The IGZO solution was spin coated at 3500 rpm for 30 s, followed by a thermal annealing step on a hot plate at 350 °C for 1 h. IGZO and passivating SU-8 films were patterned as described above.

*1.4. Electrical characterisation*

Electrical properties of the M-I-M structures (for the capacitor characterisation) were measured by Metrohm AutoLab Potentiostats/Galvanostats. In these devices, the active area (the surface in direct contact with the electrolytes) of Au and IGZO is 200 × 200 μm^2^. The TFT devices were measured by a Keithley K4200 semiconductor parameter analyzer. All measurements were performed at room temperature in ambient conditions.

**2. Cyclic voltammetry measurement**

Figure S1. Cyclic voltammetry measured in pure water and KCl ionic solution with various concentration from (a) Au/electrolyte/Au and (b) Au/electrolyte/IGZO/Au system. The scan rate is 10 mV/s.

**3. Characterisation of EGTFTs under potassium chloride (KCl) solutions**

Figure S2. Representative (a) transfer and (b) output curves of the EGTFTs operated in 1.0 M KCl solution. Statistical distribution of (c) maximum transconductance (d) threshold voltages, and (e) subthreshold swings from 10 measured devices.

**4. Impedance analysis**

Figure S3. Impedance spectra from the Au/electrolyte/Au structure. (a) Imaginary part (Z’’) vs. frequency plots for the device in KCl, NaCl, and KBr solution (1.0 M concentration) (b) Z’’ vs. frequency plots depending on KCl concentration (from 0.1 to 2.0 M).


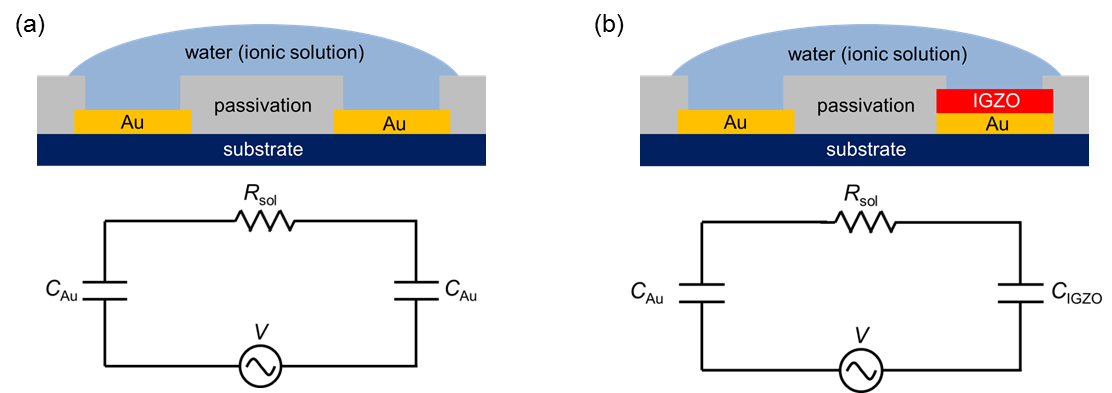


Figure S4. Structure and equivalent circuit of the coplanar-electrode devices used for the impedance measurements with (a) Au/electrolyte/Au and (b) Au/electrolyte/IGZO configuration.

Electrochemical impedance spectroscopy was conducted on two device architectures, namely Au/electrolyte/Au and Au/electrolyte/IGZO, as illustrated in Figure S4. Simple equivalent circuits are devised from the consideration of electric double layer (EDL) capacitance formed at the Au/electrolyte interface (*C*_Au_) and at the IGZO/electrolyte interface (*C*_IGZO_), as well as the solution resistance (*R*_sol_) accounting for the ionic transport. The measured small-signal impedances are expressed as total impedance (*Z*_tot_). The equation corresponding to each case is

$Z_{\text{tot}}=R_{\text{sol}}+\frac{2}{j\omega C_{\text{Au}}}$ (1),

for the Au/electrolyte/Au devices, and

$Z_{\text{tot}}=R_{\text{sol}}+\frac{1}{j\omega}\left(\frac{1}{C_{\text{Au}}}+\frac{1}{C_{\text{IGZO}}}\right)$ (2),

for the Au/electrolyte/IGZO devices.

Therefore, the *C*_Au_ values under different aqueous electrolytes are directly extracted from the imaginary part of the *Z*_tot_ measured from the Au/electrolyte/Au structures, and the *C*_IGZO_ values are deduced from the *Z*_tot_ data of the Au/electrolyte/IGZO devices with the already extracted *C*_Au_ values. In our TFT architecture, the gate/electrolyte interface area is equivalent to the tip surface dip into the liquid, and thus is much smaller than the semiconductor area in contact with the electrolyte. The gate-to-channel total capacitance under transistor action is therefore limited by the capacitance at the Au/electolyte interface. For this reason, we extract the EDL capacitance at the Au surface by analysing the impedance of the Au/electrolyte/Au systems to evaluate the channel field-effect mobilities (Table 1 in the main article).

5. Inverter circuits operation in aqueous solution

*5.1. Inverter behaviour operated in aqueous solution depending on concentration*

**Figure S5.** (a) Static behaviour of a resistor (50 kΩ) loaded inverter with IGZO EGTFTs (W/L = 200 μm/20 μm), and V_DD_ was 0.5 V. (b) Gain values extracted from resistor loaded inverter operated in pure water and KCl solution depending on various concentration (from 0.1 M to 2.0 M of KCl salt concentration in DI water)

*5.2. Signal responses depending on pulse frequency*

**Figure S6.** Signal responses of a resistor (50 kΩ) loaded inverter with IGZO EGTFTs (W/L = 200 μm/20 μm), and V_DD_ was 0.5 V with pulsed input biases (0 to 0.5 V) at (a) 1 Hz (b) 10 Hz (c) 100 Hz

*5.3. Operational stability of circuits in aqueous solution*

Figure S7. Operational stability of the inverter circuits. Output data were measured over up to 8 h of operation.

**6. Comparison of TFT parameters of reported water gated transistors**

We compared the maximum transconductance and on/off ratio of our devices to the previously reported values from the devices operated in water with various active materials, as summarized in Table S1 and Figure S16. When not specified in the original paper, transconductance values were calculated from the given saturation mobility and areal capacitance values, according to: (noted as “(Cal)” in g_m_ values)

**Table S1.** Comparison of the TFT parameters of EGTFTs operated in aqueous solutions

| **Active**  **materials** | **Electrolyte**  **materials** | **W / L (μm)** | **V_G_ (V)** | **V_D_ (V)** | **g_m,max_ (μs)** | **g_m_/W (S/m)** | **On/Off** | **Ref.** |
| --- | --- | --- | --- | --- | --- | --- | --- | --- |
| **IGZO (This study)** | **Water (Salts)** | **200 / 20** | **0.5** | **0.5** | **1150** | **5.75** | **10^8^** |  |
| **Diamond** | **PBS (KCl)** | **40 / 10** | **0.8** | **0.2** | **18** | **0.450** | **-** | **S2** |
| **Graphene** | **PBS (BSA)** | **2~5 / 2~5** | **0.1** | **0.1** | **36** | **7.2** | **<10** | **S3** |
| **Graphene/**  **Lipid bilayer** | **Water (NaF)** | **- / -** | **0.6** | **0.1** | **350** | **-** | **<10** | **S4** |
| **P3HT** | **Water** | **500 / 3** | **0.8** | **0.5** | **26.8 (Cal)** | **0.053** | **10^3^** | **S5** |
| **Rubrene** | **Water** | **180 / 14** | **1.0** | **0.5** | **0.308 (Cal)** | **0.002** | **10^4^** | **S5** |
| **Graphene** | **Water (Na_3_PO_4_)** | **40 / 16** | **1.0** | **0.1** | **420** | **10.5** | **<10** | **S6** |
| **P3HT-PMMA** | **Water** | **- / 3** | **0.5** | **0.6** | **0.41 (Cal)** | **-** | **10^4^** | **S7** |
| **P3HT** | **Water (LiClO_4_)** | **3E4 / 10** | **0.5** | **0.5** | **31.7 (Cal)** | **0.002** | **10^4^** | **S8** |
| **Pentacene** | **Water (NaCl)** | **2E4 / 40** | **0.4** | **0.1** | **1750 (Cal)** | **0.088** | **10^5^** | **S9** |
| **ZnO/HMDS** | **Water** | **2E4 / 10** | **0.8** | **0.9** | **42240 (Cal)** | **2.11** | **10^2^** | **S10** |
| **P3HT** | **Water (Saline)** | **3 / 7** | **0.5** | **0.5** | **0.012 (Cal)** | **0.004** | **10^2^** | **S11** |
| **P3HT-Biotin** | **PBS** | **1E4 / 10** | **0.6** | **0.6** | **13.1 (Cal)** | **0.001** | **10^3^** | **S12** |
| **PEDOT:PSS** | **Water (NaCl)** | **10 / 2** | **0.28** | **0.6** | **2700** | **270** | **10** | **S13** |
| **PEDOT:PSS** | **Water (NaCl)** | **10 / 5** | **1.0** | **0.6** | **2000** | **200** | **10^5^** | **S14** |
| **PEDOT:PSS** | **Cell culture**  **Medium** | **30 / 6** | **0.4** | **0.4** | **2500** | **83** | **-** | **S15** |
| **PII2T-Si** | **Water (NaCl)** | **4E3 / 50** | **1.0** | **0.6** | **4.67 (Cal)** | **0.001** | **10^3^** | **S16** |
| **ZnO** | **Water, PBS** | **6E4 / 2** | **0.5** | **0.5** | **21000 (Cal)** | **0.350** | **10^3^** | **S17** |
| **P3CPT** | **Water** | **1E4 / 2** | **0.5** | **0.5** | **225 (Cal)** | **0.023** | **-** | **S18** |
| **P3HT-COOH15** | **Water** | **1E4 / 2** | **0.5** | **0.5** | **5.25 (Cal)** | **0.001** | **-** | **S18** |
| **P3HT-co-P3PEGT** | **Water (NaCl)** | **600 / 4** | **1.0** | **0.5** | **3.9** | **0.007** | **10^2^** | **S19** |

Figure S8. Comparison of the on/off ratio versus maximum transconductance values from the previously reported results using various active thin film materials. All devices were operated in aqueous solutions.

**7. References**

1. Kim, Y.-H. *et al.* Flexible metal-oxide devices made by room-temperature photochemical activation of sol-gel films. *Nature* **489,** 128–132 (2012).

2. Dankerl, M. *et al.* Diamond Transistor Array for Extracellular Recording From Electrogenic Cells. *Adv. Funct. Mater.* **19,** 2915–2923 (2009).

3. Ohno, Y., Maehashi, K., Yamashiro, Y. & Matsumoto, K. Electrolyte-Gated Graphene Field-Effect Transistors for Detecting pH and Protein Adsorption. *Nano Lett.* **9,** 3318–3322 (2009).

4. Ang, P. K. *et al.* A Bioelectronic Platform Using a Graphene−Lipid Bilayer Interface. *ACS Nano* **4,** 7387–7394 (2010).

5. Kergoat, L. *et al.* A Water-Gate Organic Field-Effect Transistor. *Adv. Mater.* **22,** 2565–2569 (2010).

6. Hess, L. H. *et al.* High-transconductance graphene solution-gated field effect transistors. *Appl. Phys. Lett.* **99,** 033503 (2011).

7. Kergoat, L. *et al.* Use of poly(3-hexylthiophene)/poly(methyl methacrylate) (P3HT/PMMA) blends to improve the performance of water-gated organic field-effect transistors. *Org. Electron.* **12,** 1253–1257 (2011).

8. Cotrone, S. *et al.* Phospholipid film in electrolyte-gated organic field-effect transistors. *Org. Electron.* **13,** 638–644 (2012).

9. Cramer, T. *et al.* Double layer capacitance measured by organic field effect transistor operated in water. *Appl. Phys. Lett.* **100,** 143302 (2012).

10. Naim, A. A. & Grell, M. Electron transporting water-gated thin film transistors. *Appl. Phys. Lett.* **101,** 141603 (2012).

11. Schmoltner, K., Kofler, J., Klug, A. & List-Kratochvil, E. J. W. Electrolyte-Gated Organic Field-Effect Transistor for Selective Reversible Ion Detection. *Adv. Mater.* **25,** 6895–6899 (2013).

12. Suspène, C. *et al.* Copolythiophene-based water-gated organic field-effect transistors for biosensing. *J. Mater. Chem. B* **1,** 2090 (2013).

13. Khodagholy, D. *et al.* High transconductance organic electrochemical transistors. *Nat. Commun.* **4,** (2013).

14. Rivnay, J. *et al.* Organic Electrochemical Transistors with Maximum Transconductance at Zero Gate Bias. *Adv. Mater.* **25,** 7010–7014 (2013).

15. Yao, C., Li, Q., Guo, J., Yan, F. & Hsing, I.-M. Rigid and Flexible Organic Electrochemical Transistor Arrays for Monitoring Action Potentials from Electrogenic Cells. *Adv. Healthc. Mater.* n/a–n/a (2014). doi:10.1002/adhm.201400406

16. Knopfmacher, O. *et al.* Highly stable organic polymer field-effect transistor sensor for selective detection in the marine environment. *Nat. Commun.* **5,** (2014).

17. Singh, M. *et al.* Bio-sorbable, liquid electrolyte gated thin-film transistor based on a solution-processed zinc oxide layer. *Faraday Discuss* (2014). doi:10.1039/C4FD00081A

18. Toss, H. *et al.* On the mode of operation in electrolyte-gated thin film transistors based on different substituted polythiophenes. *Org. Electron.* **15,** 2420–2427 (2014).

19. Yaman, B., Terkesli, I., Turksoy, K. M., Sanyal, A. & Mutlu, S. Fabrication of a planar water gated organic field effect transistor using a hydrophilic polythiophene for improved digital inverter performance. *Org. Electron.* **15,** 646–653 (2014).
